# Oral Squamous Cell Carcinoma: Focus on Biomarkers for Screening

**DOI:** 10.30476/dentjods.2023.96159.1924

**Published:** 2024-03-01

**Authors:** Hamid Ghaderi, Mina Roshan-Zamir, Morteza Jafarinia, Estie Kruger

**Affiliations:** 1 Violet Vines Marshman Centre for Rural Health Research, La Trobe Rural Health School, La Trobe University, Bendigo, VIC, 3552, Australia; 2 Shiraz Institute for Cancer Research, Shiraz University of Medical Sciences, Shiraz, Iran; 3 Shiraz Neuroscience Research Center, Shiraz University of Medical Sciences, Shiraz, Iran; 4 Faculty of Science, School of Human Science, University of Western Australia, WA

**Keywords:** Mouth Neoplasms, Squamous Cell Carcinoma of Head and Neck, Biomarkers

## Abstract

Oral cancer is a malignant neoplasia that can originate in the oral cavity or lips. It is a serious global health problem and one of the ten most common cancers worldwide. Over the years, changes in the trends of the oral cavity and oropharyngeal cancers have been observed. The management of oral cancer is complicated due to the functional and cosmetic consequences of treating malignancies at these anatomical locations. The tumor and its treatment can affect a variety of functional activities, including smell, sight, speaking, respiration, taste, jaw function, and mastication, either temporarily or permanently. Based on the importance of this tumor, screening oral cancer for early detection and finding the best biomarkers for diagnosis is a crucial concern. In this review of literature, the etiology, risk factors, treatment, and diagnosis of oral cancer will be reviewed with a focus on the most important biomarkers.

## Introduction

Oral cancer is a serious global health issue and one of the top ten malignancies worldwide [ 1 ]. Almost 90% of this type of cancer originates from squamous cells, which is classified as oral squamous cell carcinoma (OSCC) [ 1 - 2 ]. The incidence of oral cancer is about two to three times higher among men than among women [ 3 ]. A considerable prevalence of the disease has been reported in Melanesia (Papua New Guinea) and in South-central Asia, including India, Pakistan, Sri Lanka, India, Nepal, and Bangladesh [ 4 - 5 ]. It is quite concerning that more and more young adults under the age of 30 are suffering from OSCC, according to a recent study [ 6 ]. Oral cancers are now considered a global concern due to their high incidence and low five-year survival rates. In spite of better surgical procedures and novel therapies, the survival and recurrence rates for head and neck SCC have fairly improved globally over the past few decades [ 7 ]. An increasing trend has been reported for oral cavity and pharynx cancers in the United States, about 1% per year. On average, the mortality rate of cancers of the oral cavity and pharynx has increased by 0.4% every year. For men and women, human papillomavirus (HPV)-related oral cancers increased by about 2.8% and 1.3% per year, respectively, while smoking-related oral cancers decreased by about 0.8% per year [ 8 - 9 ]. In spite of the fact that the 5-year survival rate for OSCC is around 40%, the rate could be increased up to 80% if it is detected in the early stages [ 10 ]. According to worldwide data in 2020, the number of new cases and the mortality rate for lip and oral cavity cancer were 377,713 and 177,757, respectively [ 2 , 5 ]. 

Oral cancer is a malignant neoplasia that can manifest itself anywhere in the oral cavity, lips, sinuses, or pharynx. The most common sites for the presentation of oral cancer are the floor of the mouth, lower lip, and lateral border of the tongue [ 11 - 12 ]. It has been reported that OSCC frequently arises from a pre-existing oral lesion called precursor lesions [ 13 ]. Oral precancerous lesions, such as leukoplakia and erythroplakia as the most prevalent ones, are morphologically changed tissues with malignant transformation potential [ 14 - 15 ]. The clinical appearance of oral cancer varies greatly, and the presentation in oral cavity is linked to the primary tumor. Oral cancer most frequently presents itself as an ulcerated lesion in the oral cavity, pain, or numbness in the mouth or face, or an ill-fitted denture [ 16 ]. Dry mouth, mucositis, and dysphagia are the most conspicuous symptoms of oral cancer, which appear during and after cancer treatment [ 17 ]. Hyposalivation is the most commonly known symptom in oral cancer, where a reduction in salivary gland flow causes fungal infection, altered taste, and swallowing problems [ 18 - 19 ].

The need to preserve both functional and aesthetic aspects make the management of oral cancer difficult as related to life quality, throughout the tumor resection and other treatment modalities. In addition, the tumor and its treatment can affect a variety of functions, including sight, hearing, speaking, respiration, taste, jaw movement, and mastication. Additionally, it is important to note that oral cancer is frequently detected in an advanced stage, making early diagnosis essential [ 20 ]. Therefore, screening oral cancer for early detection and finding the best biomarkers for diagnosis is a crucial concern. 

### Search Strategy

Initially, a search was conducted using the keywords “Oral Squamous Cell Carcinoma” and each of the related terms including “etiology, risk factors, treatment, diagnosis, and screening” in the title/abstract of the published findings. Then the search was narrowed down using the keyword combination “Oral Squamous Cell Carcinoma AND tumor biomarkers AND screening” to get the most relevant studies. The search was mainly through the PubMed/Google scholar/Scopus databases. For early screening, articles published in English up to 2023 were recommended. The abstracts were evaluated and the irrelevant, outdated findings and non-English studies were excluded. To make the search as comprehensive as possible, the authors incorporated all types of studies related to the subtopics. To provide additional information for the review articles, the original articles used as references were also investigated. Following the assessment of the abstracts, the full text of the selected articles was reviewed, if they met the inclusion and exclusion criteria.

## Results

As a result, 105 articles were chosen out of 132 for writing the main manuscript. There were 27 articles excluded, of which 4 articles were not written in English, 16 articles were irrelevant, and 7 articles were outdated. The remaining articles contained all required findings, especially tumor biomarkers used to screen for OSCC. A statistical analysis was not possible due to the variety of methods, populations, and study designs.

## Literature Review

This literature review intends to discuss the etiology, risk factors, screening strategies, and treatment options for OSCC, focusing on the tumor biomarkers available for early diagnosis.

### Etiology and risk factors for OSCC

Cigarette smoking habit and high alcohol intake are two major contributors to oral cancer. Snuff smoke is carcinogenic, and it can cause oral cavity and pancreatic cancer. Compared with non-smokers, smokers have a three times higher risk of developing oral cancer [ 21 ]. A smoky environment is also risky. There is an 87% greater risk of oral cancer among never-smokers who have been exposed to cigarette smoke than those never-smokers who have not been exposed [ 22 ]. The carcinogenic substances within cigarettes damage DNA, interfere with DNA repair mechanisms, and weaken the immune components in the oral cavity, which may lead to tumorigenesis [ 23 - 24 ]. For instance, cigarette smoking could promote OSCC development by activating receptor-interacting protein 2 / nuclear factor *κ*B (RIP2/NF-κB) signaling pathway and upregulating caspase-12 as a result, a factor which is involved in weakening the mucosal immunity [ 25 ]. The activation of Wnt/ mitogen-activated protein kinases (MAPK) pathways and an increase in reactive oxygen species (ROS) levels afterwards may be the cause of cancerous alterations in the epithelial cells following cigarette smoking [ 26 ]. ROS could damage DNA, therefore, evaluating the levels of DNA damage-associated proteins such as H2A histone family member X (H2AX), checkpoint kinase 2 (CHK2), and P53 in smokers might be used to estimate the risk of developing cancer [ 27 - 28 ]. Nevertheless, recent studies using the IHC method revealed a similar immunoexpression of these proteins in OSCC samples of smokers and non-smokers [ 29 ]. As a result of smoking, mucin1 (MUC1) may be overexpressed in the oral epithelial cells and localized from the superficial to basal cell layer of the oral epithelium, leading to an increased risk of developing oral epithelial dysplasia (OED) and OSCC afterwards [ 30 ]. Several events have been associated with cigarette smoke condensate (CSC)-induced tumor progression in a recent study, including miR-30a downregulation and overexpression of binding immunoglobulin protein (BiP) as an endoplasmic reticulum (ER) stress regulator that enhances vascular endothelial growth factor (VEGF) production and secretion in OSCC cells both *in vivo* and *in vitro* [ 31 ].

Alcohol is another important risk factor for oral cancer [ 32 - 33 ]. Alcohol leads to the dissolving of lipids components of the epithelium, causing epithelial atrophy and interference in DNA synthesis and DNA repair mechanisms, and increased permeability of oral mucosa. It contains genotoxic and mutagenic effects, resulting in a reduction in salivary flow. Chronic alcohol intake is linked to a disturbance in innate and acquired immunity, rendering more susceptibility to infections and cancers [ 32 - 33 ]. Recent findings revealed a possible association between heavy alcohol consumption, Toll-like receptor (TLR-9 high) tumors with reduced intratumoral CD8^+^ cells, and lower survival rates in OSCC patients [ 34 ]. Following prolonged alcohol consumption, nuclear factor of activated T cells (NFAT) signaling may be activated, increasing cancer stemness and aerobic glycolysis [ 35 ]. Other cancer-predisposing factors, including chronic irritation, exposure to other carcinogens, poor oral hygiene, viral infections including HPV, malnutrition, and genetic factors, are also suggested [ 36 ]. Genetic variation in proto-oncogenes (Myc), tumor suppressor genes (APC, p53), genes controlling normal cellular processes (EIF3E, GSTM1), and oncogene (Ras) have been suggested to play a role in the etiology of oral cancer. Other contributing factors include DNA damage repair, segregation of chromosomes, loss of heterozygosity, telomere stabilities, defects in notch signaling pathways, and regulations of cell-cycle checkpoints [ 36 - 39 ]. Lower serum vitamin D levels have been associated with an increased risk of OSCC, a lower chance of survival, and more negative chemotherapy side effects in these patients, according to a recent systematic analysis [ 40 ].

### Treatment strategies for OSCC

Treatment of oral cancers needs a delicate and careful approach to preserve adjacent tissues and organs, which is crucial to the patient’s quality of life. Generally, oral cancer can be cured, especially in those with smaller tumors and early stages, coupled with this, a reverse correlation between tumor size and the survival rate has been reported [ 41 ]. Treatment of oral cancers comprises four different modalities: surgery, radiation therapy, chemotherapy, and immunotherapy (including the use of monoclonal antibodies and immune checkpoint inhibitors) [ 42 ]. Depending on the stage and extension of the tumor, possible side effects, and the patient's overall health, one or a mixture of these will be applied. However, surgery is the first option in most cases, a well-accepted treatment for most oral cancers. Following tumor resection, the rehabilitation and reconstruction of affected surrounding tissues are essential to maintain the aesthetics and quality of life. Surgery is the tumor’s excision with some healthy surrounding tissue, called a safe margin (SM). The extension of the SM varies, depending on the anatomic location and tumor invasion. The most common surgical procedures implemented in oral cancer include glossectomy, mandibulectomy, maxillectomy, and neck dissection [ 43 - 44 ]. 

### Screening for OSCC

**Figure 1:**
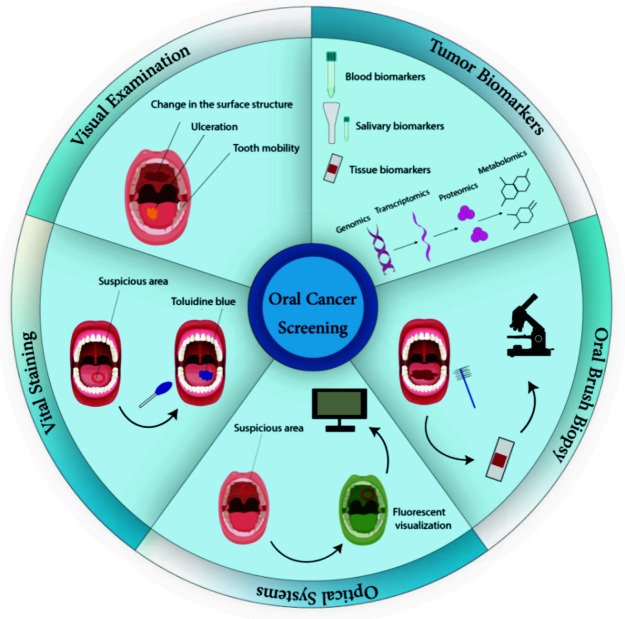
Steps in oral squamous cell carcinoma (OSCC) screening, from a routine visual examination to a sophisticated molecular detection

Early diagnosis is a critical element in reducing the mortality rate of oral cancers. According to the tumor node and metastasis (TNM) classification, tumor size plays a crucial role in the mortality and morbidity rate. Unfortunately, in most countries, many patients with oral cancer are diagnosed with advanced disease (stages III/IV) with multiple metastases. As a result, the five-year survival rate for stage I oral cancer is over 80%, compared with roughly 20% for advanced stages. Consequently, oral screening is regarded as the best strategy to reduce patients’ mortality and morbidity rates, especially among high-risk individuals [ 45 - 46 ]. In the case of oral cancer, screening deals with symptomatic patients with none-healing suspicious lesions, a normal population identifying early changes in oral epithelium and oral abnormality associated with dysplastic features [ 47 ]. Different screening strategies for OSCC have been illustrated in Figure 1.

### Diagnostic value of biopsy for OSCC

For many years, routine oral examination has been the primary means of detecting and diagnosing oral malignancies. Biopsy and histopathological analysis, as the gold standards for OSCC diagnosis, will further be prescribed for suspicious findings in clinical examinations. Although histopathology provides a definitive diagnosis for oral cancers, it is a sensitive technique that requires several days of processing for clinicians to obtain results. Based on the pathological findings, severe dysplasia extending beyond the epithelium and invasion of the underlying lamina propria is regarded as carcinoma. Although dysplasia in histopathology is regarded as an elevated risk of malignant transformation, it is a snapshot of the current situation, which will not predict future malignant transformation [ 48 ]. Based on this, premalignant lesions and those confirmed with dysplastic changes must be observed clinically and require multiple biopsies to detect any changes over time.

A perfect specimen must have sufficient size that includes both the suspected lesion and normal surrounding tissue. A biopsy sample must include tissues with the greatest dysplastic signs and represent the significant changes in epithelium. As a case in point, those areas with a sign of induration, ulcer, and reddish demonstration are the potential sites to collect the biopsy. Other factors should be considered to improve histopathological diagnosis, including access to vital clinical information and surgical findings for the pathologist [ 49 ]. Finally, a knowledgeable and expert oral pathologist plays a mandatory role in the biopsy’s outcome. Overall, histopathology observes changes at the cellular level, while further detection of molecular changes requires extra specific tools.

### Visual examination

Among several screening methods, visual examination is a fundamental method to discover clinical changes in oral epithelium, including ulceration, tooth mobility, and changes in the surface texture. Reports indicated that visual examination is the most readily available method to detect precancerous lesions, especially in individuals at high risk of oral cancers, together with heavy drinkers and smokers [ 50 ]. 

Although the visual examination is a cheap and accessible oral examination method, it does not provide strong sensitivity and specificity for diagnosing oral lesions. Therefore, other complementary methods have been introduced to help clinicians detect early changes and define the lesions' nature [ 51 - 52 ]. Currently available techniques are Toluidine blue staining, micro-brush cytology, biomarkers, and optical techniques [ 46 ]. Below, some of the most common methods will be covered. 

### Vital staining

Implementation of toluidine blue (TB) staining before a biopsy is a valuable and inexpensive way to highlight suspected malignant areas. Malignant cells have elevated nuclear activity with higher DNA content. TB binds to abnormal cells' nucleus component with higher intensity because of their high affinity for nucleic acid [ 53 ]. Therefore, it is considered a non-invasive method that highlights the lesion, assisting the clinicians in collecting an accurate biopsy [ 54 ]. It has been reported that TB as a simple and accessible method is recommended to screen the high-risk population, detecting malignant and premalignant lesions more potent than the conventional oral examination (96.7% and 40% sensitivity, respectively). The presence of blue dye with no further confirmation of carcinoma (false positive) occurs in 8-10% of cases, while the false-negative result is usually rare [ 55 ]. One of the advantages of the vital staining screening methodology is that this method requires no dentist/ specialist and it can be performed at the primary dental care level by any experienced oral healthcare provider.

### Optical systems

In the past few years, new technology was introduced in which suspicious tissues are exposed to an external light source, exciting certain amino acids, metabolic products, and structural proteins inside the tissue, creating visible and quantifiable light. Needless to say, in oral epithelium abnormalities, the emission rate is interpreted as changes in cellular morphology. Changes in the intensity of color by an exogenous source distinguish normal from abnormal tissues [ 56 - 57 ]. One of the recently introduced optical systems is fluorescent visualization, a non-invasive and repeatable method. In this system, the normal and the precancerous mucosa are visualized differently [ 58 ]. The high false-positive result rate is reported to be one of the disadvantages of optical systems [ 59 ]. There have also been arguments made in the literature about the utility of optical systems, detecting oral lesions superior to the oral examination. A more comprehensive cohort study is required to validate these assertions. 

### Oral brush biopsy

Cytology brush was first introduced in 1963, but it was not accepted among clinicians because of its low sensitivity and specificity to detect dysplastic and malignant lesions [ 60 ]. Advancements in this technology have provided an excellent opportunity to employ this approach as a conventional screening program. Implementation of the cytology brush in oral lesion detection was followed by its successful application in cervical cancer [ 61 ]. Patients are more receptive to this approach, because the oral cytology brush (OCB) is a safe and minimally invasive procedure to collect oral mucosa cells. Therefore, it is regarded as a promising approach for screening and early diagnosis of oral premalignant lesions [ 60 ].

Several reasons have been reported for poor sensitivity and false negative results from OCB. To exemplify, obtaining samples from a superficial rather than a full-thickness layer, the presence of blood/debris, and other obscuring factors were mentioned [ 60 , 62 - 63 ]. Combining liquid base cytology (LBC) with conventional OCB has provided greater diagnostic accuracy to detect abnormal tissues [ 64 ]. In this method, mucosal cells are harvested by a plastic device and then kept in a preservative medium until transferred to the laboratory [ 65 ]. Although several studies supported using oral cytology combined with LBC, a longitudinal cohort study with a large sample size must be conducted to validate the efficacy and accuracy of this method [ 64 , 66 ].

### Tumor biomarkers

The development of immunological techniques has led scientists to look for changes at the cellular and molecular levels in cancers. Detection of abnormal gene/protein expression in biological specimens has opened various windows to the world of tumor biomarkers for cancer screening. As an instance, prostate-specific antigen (PS-A) for prostate cancer, cancer antigen 125 (CA-125) for ovarian cancer, and carcinoembryonic antigen (CEA) and cancer antigen 19-9 (CA 19-9) for gastrointestinal cancers have long been used in cancer screening [ 67 ].

Biomarkers are generally categorized at the level of metabolomics, proteomics, and genomics. Oral cancer molecular biology and oncology research focus on key biological markers or molecules that can contribute to risk assessment, cancer formation, recurrence prediction, screening, invasion/metastasis, prognosis, and monitoring cancer therapy response [ 68 ].

### Salivary/Serum (Plasma)/ Tissue biomarkers

Saliva has been indicated as an alternative medium for screening oral cancer since it is non-invasive, inexpensive, and easily accessible to collect [ 69 ]. Saliva contains several chemokine/cytokine and exfoliated cells, which allows not only for genetic changes to be assessed but is also a powerful search tool for protein biomarkers in individuals with a high risk of developing oral cancer [ 62 ]. In recent years, saliva has been subjected to proteomics technology to find new biomarkers for oral cancers [ 70 ]. It appears that salivary biomarkers such as mRNA and miRNA estimated through the polymerase chain reaction (PCR) have a good screening potential for early detection of OSCC, but further research will be required in order to confirm these findings [ 71 ]. According to a recent systematic and meta-analysis review, salivary mRNA biomarkers including dual specificity 1 protein (DUSP-1) and calcium-binding protein S100P demonstrated the highest specificity and sensitivity for early detection of OSCC (91%) [ 72 ]. Recent advancements in the identification of salivary biomarkers for OSCC using transcriptomic, proteomic, and metabolomics strategies have been mentioned in Tables 1-3 respectively. Using gas chromatography mass spectrometry (GC/MS) for metabolomics analysis, it was discovered that glucose, cysteine+cysteine, nonanoicic acid, and galactose might all be used as possible biomarkers for OSCC screening [ 73 ]. 

**Table 1:** Oral squamous cell carcinoma (OSCC) biomarkers identified through transcriptomic analysis of saliva (for early screening)

Biomarker	Result	AUC, Specificity, Sensitivity	References
	Overexpressed in OSCC patients compared to HCs	0.7659, 0.682, 0.814	[ 79 ]
miR-345-3p		0.6924, 0.886, 0.488	
miR-31-5p	Decreased in OSCC patients compared to HCs	0.7326, 0.818, 0.605	
miR-424-3p		Combination of three: 0.87, 0.77, 0.86	
HGF, VEGF, PIGF, MMP-1, MMP-3, MMP-8, MMP-9, MMP-10, MMP-13, and TIMP-2	Upregulated in OSCC patients compared to the CG	Not mentioned	[ 80 ]
cfDNA integrity indexes:	Upregulated in OSCC patients compared to the CG		[ 81 ]
ALU115/ALU60		0.8211, 73.33%, 83.33%	
ALU247/ALU60		0.7018, 73.33%, 83.33%	
CCL20	Overexpressed in OSCC patients compared to HCs	0.979, 0.980, 1.000	[ 82 ]
miR-15a and miR-16-1	Downregulated in OSCC patients compared to HCs	90%, 86.67%, 93.3%	[ 83 ]
		93.3%,92.33%, 86.67%	
CPLANE1	Overexpressed in OSCC patients compared to OPMDs patients and HCs	Not mentioned	[ 84 ]
miR-106b-5p, miR-423-5p and miR-193b-3p	Differentially expressed in OSCC patients compared to HCs	0.813, 0.731, 0.842	[ 85 ]
		0.851, 0.639, 0.885	
		0.748, 0.639, 0.750	
		Combination: 0.98, 0.942, 0.974	
miR-30c-5p	Downregulated in OSCC patients compared to HCs	0.82, 74%, 86%	[ 86 ]
microRNA-200a and microRNA-134	Increased in OSCC patients compared to the smoker and the CG	Not mentioned	[ 87 ]
IL-1β & IL-8			
NUS1 and RCN1	Overexpressed in OSCC patients compared to HCs	0.715, 0.707, 0.683	[ 88 ]
		0.759, 0.900, 0.683	
miR-24-3p	Overexpressed in OSCC patients compared to HCs	0.738, 0.800, 0.644	[ 89 ]
LDOC1 (a tumor suppressor gene)	Upregulated in females and downregulated in males with OSCC compared to HCs	Not mentioned	[ 90 ]
IL-6 mRNA	Upregulated in OSCC patients compared to the CG	0.9379, 0.819, 0.945	[ 91 ]
miR-31 and miR-21	Upregulated in OSCC patients compared to controls Downregulated in OSCC patients compared to controls	0.95	[ 92 ]
miR-31 and miR-21			
miR-512-3p	Overexpressed in extracellular vesicles of OSCC patients compared to the CG	0.847, high sensitivity and specificity	[ 93 ]
miR-412-3p		0.871, high sensitivity and specificity	

Healthy controls (HCs); Control group (CG); Area under curve (AUC); Vascular endothelial growth factor (VEGF); Placental growth factor (PIGF); Hepatocyte growth factor (HGF); Matrix metalloproteinase (MMP); Tissue inhibitor of metalloproteinases 2 (TIMP2); cell-free DNA (cfDNA); C-C motif chemokine ligand 20 (CCL20); Ciliogenesis and planar polarity effector 1 (CPLANE1); Oral potentially malignant disorders (OPMDs); Nuclear undecaprenyl pyrophosphate synthase 1 (NUS1); Reticulocalbin 1 (RCN1); Leucine Zipper, Down-regulated in Cancer-1 (LDOC1)

**Table 2:** Oral squamous cell carcinoma (OSCC) biomarkers identified through proteomic analysis of saliva

Biomarker	Result	Potential use	AUC, Specificity, Sensitivity	References
IL-1β, IL-6, IL-8	Increased in OSCC patients compared to the CG	Early detection	0.724, 84%, 64%, 0.856, 96.6%, 75.6%, 0.978, 96.7%, 96.9%	[ 94 ]
A set of autoAbs to LMAN2, PTGR1, RAB13, and UQCRC2	Increased in OSCC patients compared to the CG	Early diagnosis	Not mentioned	[ 95 ]
Cathepsin B	Increased in OSCC patients compared to the control group and associated with well differentiated OSCC	Diagnosis and monitoring of OSCC	83%, 80%, 85%	[ 96 ]
Non-apoptotic tumoral cell-secreted microvesicles (MVs)	Higher levels in OSCC patients with T4 and T3 tumor stages compared to those with T2 and T1 and HCs	Progressive marker of OSCC	Not mentioned	[ 97 ]
MMP-9	Increased in OSCC patients compared to the controls/ Decreased post-surgery of OSCC	Diagnosis and prognosis	0.96, 100%, 89.6%	[ 98 ]
MMP-12	Increased in OSCC patients compared to HCs	Early diagnosis	100%, 100%, 100%	[ 99 ]
AHSG and KRT6C	Upregulated in OSCC patients compared to the controls	Diagnosis	82.4%, 73.5%, 785	[ 100 ]
KLK1, BPIFB2, LACRT and AZGP1	Downregulated in OSCC patients compared to the controls			
MLT	Increased in OSCC patients compared to HCs	Diagnosis	0.841, 57.6%, 97.1%	[ 101 ]
CD44, S100A7, and S100P	Increased in OSCC patients compared to HCs	Early detection	0.712, 54.55, 91.67	[ 102 ]
			0.744, 72.73, 81.82	
			0.76, 72.73, 81.82	
IL-1β	Increased in OSCC patients compared to HCs	Early screening/ post-treatment follow-up	0.9017, 59.5%, 71%	[ 103 ]
IL-8			0.7619, Not mentioned, 63.8%	
LGALS3BP			0.7296 (LGALS3BP discriminates between PMODs and controls)	
MMP-9	Increased in OSCC patients compared to the CG/ Higher MMP-9 levels in poorly differentiated OSCC group	Diagnosis and follow-up	0.917, 59%, 100%	[ 104 ]
MMP1, PADI1, TNC, CSTA and MMP3	Significant changes in the levels/ an elevated disease-discriminating power	OSCC detection	AUC: 0.914, 0.827, 0.813, 0.77, and 0.753	[ 105 ]
IL6 protein and mRNA	Elevated in OSCC patients compared to controls	Diagnosis	Not mentioned	[ 106 ]
MMP-1, MMP-2, MMP-10, MMP-12, metalloprotease 9, cathepsin V, kallikrein 5, ADAM9, and ADAMST13	Increased in OSCC patients compared to patients with other oral diseases and HCs	Early screening	Combination of ADAM9/Cathepsin v/Kallikrein 5: 0.938, 0.9917, 0.9	[ 107 ]
SNCG	Increased in OSCC patients compared to controls	Diagnosis	0.865, 68.7%, 97.5%	[ 108 ]
NID1	Increased in OSCC patients compared to HCs and associated with poor prognosis	Diagnosis and prognosis	0.714	[ 109 ]
IL-10	Increased in OSCC patients compared to the HCs but decreased after tumor removal, Decreased in OSCC patients compared to the normal subjects but increased after tumor removal	Monitoring response to tumor treatment	Not mentioned	[ 110 ]
IFN-γ				
ET-1	Increased in OSCC patients compared to the HCs	Diagnosis	Not mentioned	[ 111 ]
FGA, CFH, and SERPINA1	Overexpressed in OSCC patients compared to HCs	Early detection, prognosis	0.740, 87%, 51.9%	[ 112 ]
			0.661, 95%, 37.7%	
			0.740,79%, 64.9%	
			Combination: 0.751	
bFGF	Increased in OSCC patients compared to the CG	Early screening	Not mentioned	[ 113 ]
PRDX-2, ZAG	Increased in OSCC patients compared to CFCs, Upregulated in lesion cells compared with oral exfoliated cells	Early screening	Combination: 0.999, 98.77%, 100%	[ 114 ]
Naa10p and CEA	Increased in OSCC patients compared to patients with OPMLs and HCs	Use of the combination of both for early detection	0.884, 83.3%, 81.1%	[ 115 ]
			0.875, 81.7%, 80.2%	
			Combination: 0.944, 85%, 92.5%	
IL-1β, IL-6, IL-8, MIP-1β, eotaxin, IFN-γ, and TNF-α	Increased in OSCC patients compared to HCs	Early detection	0.729, 79.17%, 60.98%, 0.823, 70.83%, 82.93%, 0.783, 79.17%, 65.85%, 0.681, 79.17%, 58.545, 0.662, 65.50%, 70.73%, 0.657, 50%, 80.49%, 0.749, 100%, 39.02%	[ 116 ]
LDH and CYFRA 211	Increased in OSCC patients compared to controls	Early detection	Not mentioned	[ 117 ]
SLC3A2, S100A2	Increased in OSCC patients compared to controls	Early diagnosis	Combination: 0.89, 83.33%, 83.33%	[ 118 ]
IL1RN	Decreased in OSCC patients compared to controls			
TNF-α	Increased in OSCC patients compared to controls	Prediction of OSCC	0.992,93.3%, 93.3%	[ 119 ]
AKR1B10	Increased in OSCC patients compared to controls and associated with poor prognosis	Screening and monitoring	Not mentioned	[ 92 ]

Healthy controls (HCs); Control group (CG); Area under curve (AUC); Lectin, Mannose Binding 2 (LMAN2); Prostaglandin Reductase 1 (PTGR1); Matrix metalloproteinase (MMP); Alpha 2-HS Glycoprotein (AHSG); Kallikrein 1 (KLK1); Lacritin (LACRT); Alpha-2-Glycoprotein 1, Zinc-Binding (AZGP1); Peptidyl arginine deiminase, type I (PADI1); Tenascin C (TNC); Cystatin-A (CSTA); A disintegrin and metalloprotease (ADAM); A disintegrin and metalloprotease with thrombospondin type 13 motifs (ADAMST13); complement factor H (CFH); fibrinogen alpha chain (FGA); synuclein-γ (SNCG); nidogen-1 (NID1); S100 calcium-binding protein A2 (S100A2); Endothelin-1 (ET-1); alpha-1-antitrypsin (SERPINA1); basic fibroblast growth factor (bFGF); Peroxiredoxin-2 (PRDX-2); Zinc-alpha-2-glycoprotein (ZAG); N-α-acetyltransferase 10 protein (Naa10p); Carcinoembryonic antigen (CEA); oral premalignant lesions (OPMLs); Lactate Dehydrogenase (LDH); ); potentially malignant disorders (OPMDs); solute carrier family 3 member 2 (SLC3A2); interleukin-1 receptor antagonist protein (IL1RN); tumor necrosis factor-alpha (TNF-α); Aldo-keto reductase family 1 member B10 (AKR1B10); Melatonin (MLT)

**Table 3:** Oral squamous cell carcinoma (OSCC) biomarkers identified through metabolomic analysis of saliva

Biomarker	Result	Potential use	AUC, Specificity, Sensitivity	References
3-methylhistidine	Higher levels of 3-methylhistidine associated with lower overall survival rate	Significant prognostic factor of overall survival in OSCC patients	HR=1.711, *p* value=0.048	[ 120 ]
Decanedioic acid, 2-methyloctacosane, eicosane, octane, 3,5-dimethyl, pentadecane, hentriacontane, 5,5-diethylpentadecane, nonadecane, oxalic acid, 6-phenylundecanea, l-proline, 2-furancarboxamide, 2-isopropyl-5-methyl-1-heptanol, pentanoic acid, docosanemetabolites	Differed significantly between control, oral leukoplakia and OSCC	Early detection of OSCC and oral leukoplakia	Not mentioned	[ 121 ]
Malic acid, maltose, methionine, inosine	Upregulated in OSCC patients compared to HCs	Early diagnosis	AUC > 0.8	[ 122 ]
1-methylhistidine, inositol 1,3,4-triphosphate, d-glycerate-2-phosphate, 4-nitroquinoline-1-oxide, 2-oxoarginine, norcocaine nitroxide, sphinganine-1-phosphate, and pseudouridine	Upregulated in OSCC patients compared to the CG	Early diagnosis	Not mentioned	[ 123 ]
GGT	Increased in OSCC patients compared to patients with normal oral cavity findings	Early detection	Not mentioned	[ 124 ]
Malondialdehyde	Increased in OSCC patients compared to the controls	Early diagnosis	1.000, 100%, 100%	[ 125 ]
Nitric oxide			1.000, 100%, 100%	
Salivary albumin levels	Increased in OSCC patients compared to HCs	Further studies are required to use these markers as diagnostic biomarkers	Not mentioned	[ 126 ]
Salivary uric acid levels	Decreased in OSCC patients compared to HCs			

Healthy controls (HCs); Control group (CG); Area under curve (AUC); Hazard ratios (HRs); Gamma-glutamyl transpeptidase (GGT)

Tables 4 and 5 provide an overview of recent advancements in identifying serum (plasma) and tissue biomarkers for OSCC. Additionally, biomarkers can be utilized to identify therapeutic targets and monitor the effectiveness of treatment. In this regard, Monteiro *et al*. [ 74 ] have demonstrated that the overexpression of a mammalian target of rapamycin (mTOR) protein can be considered a potential therapeutic target in individuals diagnosed with OSCC. Yang *et al*. [ 75 ] have also shown that growth differentiation factor 15 (GDF15) expression might be implemented as a prognostic and predictive marker for patients undergoing induction treatment with cisplatin, docetaxel, and 5fluorouracil (TPF). Some biomarkers detect germline mutations that are effective in predicting individuals at high risk of cancer development. These biomarkers are involved in the cell cycle, apoptosis, and cancer risks, such as polymorphism in p53/p73, murine double minute 2 (MDM2), cyclin D1 (CCND1), and heavy Ras (H-Ras) [ 76 ]. Another implication of biomarkers in oral cancer is to detect probable recurrence in patients who have had adjuvant treatment.

**Table 4:** Serum (plasma) biomarkers of oral squamous cell carcinoma (OSCC)

Biomarker	Type of biomarker	Result	Potential use	AUC, Specificity, Sensitivity	References
miR-92a-3p, miR-92b-3p, miR-320c and miR-629-5p	Transcriptomic (Serum levels)	Upregulated in OSCC patients compared to controls/ Decreased after surgery but increased following recurrence	Diagnosis and monitoring	0.7108, 0.9333, 0.4348	[ 127 ]
				0.7269, 0.4667, 0.913	
				0.8206, 0.9556, 0.6957	
				0.7011, 0.6222, 0.7391	
				Combination: 0.899, 0.978, 0.739	
miR-130a	Transcriptomic (Plasma-derived exosomal miRNAs)	Increased in OSCC patients compared to HCs/ Associated with higher tumor stages	Diagnosis and prognosis	0.812,45.7%, 98.5%	[ 128 ]
miR-138 and miR-424-5p	Transcriptomic (Serum levels)	Decreased and increased in OSCC patients compared to controls, respectively	Early detection	Not mentioned	[ 129 ]
AC007271.3 (a type of long non-coding RNA), SCCA, TSGF	Transcriptomic (Serum levels)	Differentially expressed in OSCC patients compared to controls	Early diagnosis	0.873, 84.5%, 77.6%	[ 130 ]
				0.719, 93.3%, 55.0%	
				0.648, 66.7%, 63.3%	
				Combination: 0.917, 93.1%, 80%	
30 miRNAs	Transcriptomic (Serum levels)	Differentially expressed in OSCC serum compared with normal controls	A biomarker of OSCC progression	Not mentioned	[ 131 ]
miR-222-3p, miR-150-5p, and miR-423-5p	Transcriptomic (Plasma levels)	Differentially expressed in patients with OSCC, oral leukoplakia, and normal controls	Early detection	0.520,87.14%, 23.85%	[ 132 ]
				0.702,77.14%, 60.55%	
				0.677,72.86%, 58.72%	
				Combination: 0.749	
IP-10 Eotaxin, G-CSF, and IL-6	Proteomic (Plasma levels)	Increased in OSCC patients compared to the CG	Early detection Tumor progression	0.793, 70.83, 78.05	[ 116 ]
		Increased in stages III/IV compared to stages I/II			
C-reactive protein, Carbonic anhydrase-1, and Fibronectin	Proteomic (Plasma proteome analysis)	Plasma proteins can be used as OSCC biomarkers	Putative biomarkers of OSCC	Not mentioned	[ 133 ]
HSP90α	Proteomic (Serum levels)	Upregulated in the serum of OSCC patients compared to controls	Predictive marker	Not mentioned	[ 134 ]
MCSF, I309, MMP3 and CTACK, AXL, GDF15	Proteomic (Serum levels/ Protein microarray analysis)	Elevated in OSCC patients compared to HCs	Diagnosis	0.938, 0.889, 0.833	[ 135 ]
				0.951, 0.889, 0.833	
				0.969, 0.999, 0.833	
				0.907, 0.999, 0.778	
				0.914, 0.889, 0.778	
				0.957, 0.999, 0.778	
Decanoylcarnitine, cysteine and cholic acid	Metabolomic (Plasma)	Differential metabolites	Diagnosis	0.905, 80.2%, 94%	[ 136 ]
				0.966, 97.9%, 90%	
				0.965, 93.7%, 98%	
				Combination: 0.998, 97.9%, 98%	
Albumin and uric acid	Metabolomic (Serum levels)	Decreased in OSCC patients compared to HCs	Further studies are required to use these markers as diagnostic biomarkers	Not mentioned	[ 126 ]
Sphingolipids	(Plasma levels)	17 sphingolipids decreased in OSCC patients compared to controls	Diagnosis and prognosis	Not mentioned	[ 137 ]

Healthy controls (HCs); Control group (CG); Area under curve (AUC); Squamous cell carcinoma antigen (SCCA); tumor-specific growth factor (TSGF); Interferon gamma-induced protein 10 (IP-10); Granulocyte colony-stimulating factor (G-CSF); heat shock protein alpha (HSP90α); macrophage colony-stimulating factor (M-CSF); Matrix Metallopeptidase 3 (MMP3); Growth Differentiation Factor 15 (GDF15)

**Table 5:** Tissue biomarkers of oral squamous cell carcinoma (OSCC)

Biomarker	Type of biomarker	Result	Potential use	AUC, Specificity, Sensitivity	References
HGF, VEGF, PIGF, MMP-1, MMP-3, MMP-8, MMP-9, MMP-10, MMP-13, and TIMP-2	Transcriptomic	Upregulated in OSCC patients compared to the CG	Diagnosis and prognosis	Not mentioned	[ 80 ]
PLCE1	Transcriptomic	Higher levels in OSCC tissues compared to adjacent normal tissues/ Associated with poor prognosis	Diagnosis and prognosis	0.865,78.8%, 75.8%	[ 138 ]
DDX59-AS1 (a lncRNA)	Transcriptomic	Overexpressed in OSCC tissue compared to the normal tissue /Higher levels associated with poor prognosis	Diagnosis and prognosis	0.732	[ 139 ]
SMAD7	Transcriptomic	Upregulated in OSCC patients compared to the normal tissues	Diagnosis	Not mentioned	[ 140 ]
hsa_circ_0086414	Transcriptomic	Downregulated in OSCC tissues compared to adjacent healthy tissues	Diagnosis	0.749,87.3%,65.5%	[ 141 ]
miR-3651	Transcriptomic	Downregulated in OSCC tissues compared to normal mucosa	Diagnosis	0.78,70.3%,76.1%	[ 142 ]
CXCR7	Transcriptomic	Overexpressed in tumor endothelial cells compared to normal endothelial cells and associated with higher cancer stage	Diagnosis and prognosis	Not mentioned	[ 143 ]
LINC01697, LINC02487, LOC105376575, AC005083.1, SLC8A1-AS1, and U62317.1	Transcriptomic	Differentially expressed between OSCC patients and normal oral tissues	Diagnosis	0.995,88.9%,98.2%	[ 144 ]
48 miRNAs	Transcriptomic	Differentially expressed in tumor tissues compared with normal tissues Upregulated in cancerous tissues	A biomarker of OSCC progression	Not mentioned	[ 131 ]
hsa-miR-32-5p					
Sphingolipids		4 sphingolipids elevated in OSCC patients compared to controls	Diagnosis and prognosis	Not mentioned	[ 137 ]

Control group (CG); Area under curve (AUC); Vascular endothelial growth factor (VEGF); Placental growth factor (PIGF); Hepatocyte growth factor (HGF); matrix metalloproteinase (MMP); Phospholipase C epsilon1 (PLCE1); smad family member 7 (SMAD7); Chemokine receptor 7 (CXCR7)

Sulzyc-Bielicka *et al*. [ 77 ] showed that patients with increased thymidylate synthase (TS) expression are at a higher risk of early recurrence of oral cancer in the post-treatment interval. Regarding the use of biomarkers to detect invasion, metastasis, and monitor therapeutic response in patients with metastatic carcinoma, Huang *et al*. [ 78 ] identified miRNA-459-5p and G-protein-coupled receptor kinase-interacting protein 1 (GIT1) as potential biomarkers for the invasion and metastatic phenotypes in OSCC, and their expression levels are inversely correlated.

## Conclusion

Oral cancer is a major public health concern, currently ranked among the top ten global challenges. Therefore, the availability of powerful screening tools for early detection is a core question. The biomarkers could target different parts of the body, especially the saliva and the oral cavity. Considering the value of biomarkers for early diagnosis, prognosis, and recurrent potential in post-treatment, the development, and discovery of new biomarkers is still a work in progress. It is expected that advancements in new high-throughput technologies such as proteomics and collection and evaluation of the big RNA-seq data introduction of new and reliable bio-markers for OSCC will become a reality in the not-too-distant future.
